# Identification of beagle food taking patterns and protocol for food effects evaluation on bioavailability

**DOI:** 10.1038/s41598-018-30937-1

**Published:** 2018-08-24

**Authors:** Guoqing Zhang, Caifen Wang, Li Wu, Jian Xu, Xiaoxiao Hu, Shailendra Shakya, Yuanzhi He, Xiaohong Ren, Weidong Chen, Jiwen Zhang

**Affiliations:** 1 Institute of Drug Metabolism, School of Pharmaceutical Sciences, Anhui University of Chinese Medicine, Hefei, China; 2 Center for Drug Delivery Systems, Shanghai Institute of Materia Medica, Chinese Academy of Sciences, Shanghai, China; 3 School of Pharmacy, Key Laboratory of Molecular Pharmacology and Drug Evaluation, Ministry of Education, Yantai, China; 4 University of Chinese Academy of Sciences, Beijing, China

## Abstract

Food is a known primary role to the exposure of the drugs orally administered. Since each animal may have unique food taking pattern and it is difficult to manipulate the food taking to animals, there lacks rationalized protocol for the food effects in pre-clinic study. The objective of this study was to identify the beagle food taking patterns and demonstrate their effects on bioavailability in valsartan. Herein, four types of food taking patterns of beagle were identified via inter-day and intra-day analysis, and named as Persisting, Pulsing, Postponing, Pushing (“4P Modes”), respectively, which were also validated by principal component analysis (PCA). Interestingly, food intake resulted in a reduced area under the concentration-time curve (AUC_0–12h_), maximum concentration (C_max_) and absorption rate, whilst the reduction varied in “4P Modes” of food taking. General considerations in the design of experiment for food effect to the bioavailability in beagles have been established as: to recognize the food taking patterns in each animal, to confirm the inter-day stability of the food taking behaviors, to trace the food taking patterns in parallel with plasma sampling. In conclusion, the right animals with proper food taking patterns should be assessed and selected for pre-clinic bioavailability evaluations.

## Introduction

Food and drug interaction has been a complicated topic that deserves more attentions during drug research and new formulation development. Food can affect the absorption, metabolism, excretion and other processes of drugs in gastrointestinal (GI) tract, which will alter the bioavailability in comparison with that in empty stomach^1^. In huge number of the past studies, the bioavailabilities of many drugs orally administered were affected by food, such as omecamtiv mecarbil^2^, fosamprenavir^3^, dichloroacetate^4^, propranolol^5^, nifedipine^6^, veliparib^7^ and odanacatib^8^. Foods alter the bioavailabilities of the drugs depending on multiple factors, including the alterations in gastric emptying time, bile flow, GI pH, luminal metabolism and physico-chemical interaction between foods and the dosage form^9^. Foods also undergo complexation with drugs by biochemical or physical interaction, such as hydrolysis, oxidation, neutralization and precipitation, to influence the drug absorption^10^. Particularly, food could increase in viscosity with drugs that hinders the drug diffusion into mucosal surface leading to reduced drug absorption^11^. Furthermore, the stability of drugs also relies on the physical and chemical environment in GI tract.

Therefore, the food effects to drug bioavailability should be investigated systematically in fed and fasted states to guide clinical rational use of drug especially for chronic administration, and to provide information on the control of the determination of drug dosage or the meal before and after drug administration^12,13^. The FDA has provided guidance on how to conduct a reasonable study according to the food effect on humans, which is treated with a randomized, balanced, single-dose, two-period, cross-over design for the food effect research on oral bioavailability under fasted and fed treatment^14,15^. In order to amplify the effect of food on oral bioavailability, a special test meal that contains high-fat, high-calorie and reasonable time of administration are always chosen in the fed group^16^. Though the animal experiment to assess the bioavailability and bioequivalence is an important section in preclinical research, there is yet no official guidance on animal bioavailability test on fed and fasted condition, thus some reports just simply follow protocol on humans models.

The beagle is one of the primary preclinical animal models for researches of pharmacokinetics and the effect of food in drug absorption, because of the easy administrations of various dosage forms and test meals^17,18^. However, the subjective initiative of beagle is lower than the human being, thus it is difficult to control consumption of food before the pharmacokinetic evaluations. For instance, 400 g of canned dog food as standard food with 2.5% fat and 7.5% protein has been studied^19^. However, it is without any investigation and interpretation for details like the amount of consumptive food of beagles, of which may has potential effect for the results. Furthermore, it is commonly known that the feeding is relatively free for beagles, but the food taking patterns of beagle are definitely not regular.

Valsartan is an angiotensin II receptor blocker which is rapidly absorbed from GI tract after oral administration for treatment of hypertension^20,21^. It was reported that there was significant food effects on its absorption when valsartan tablets/capsules monotherapies are administered with food in human subjects, of which C_max_ and AUC decreased by 50% and 40%, respectively^22^. Therefore, valsartan capsules were used as a model drug in this study to demonstrate the influence of food taking patterns on drug absorption in beagles. On the other hand, plasma concentrations of valsartan were measured using high-performance liquid chromatography-tandem mass spectrometry (HPLC-MS/MS) and pharmacokinetic parameters were analyzed by non-compartmental model. The information about the beagles feeding behavior was acquired in 7 consecutive days and in the same period of pharmacokinetics experiment. Then, the food taking patterns were classified based upon the food intake using method of principal component analysis (PCA). The identification of food taking patterns of beagle helps to select the right subjects for experiments and to standardize the bioavailability and bioequivalence tests in view of the animals’ food intake characteristics.

## Results

### Identification of food taking patterns of beagle

**Figure 1:**
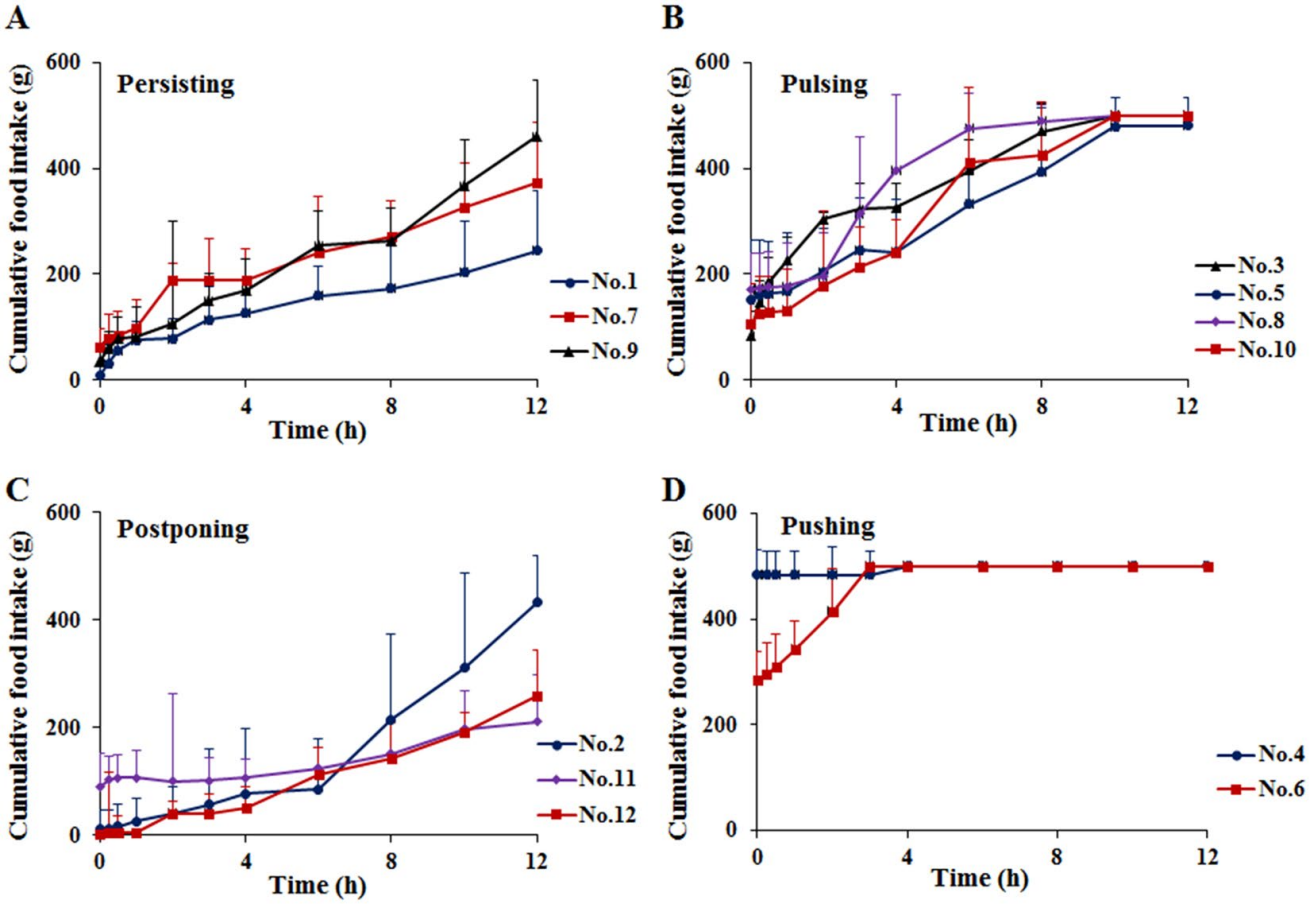
Four types of food taking were identified and named as Persisting, continuous slow food intake within 12 h. (**A**) Pulsing, taking food in particular periods. (**B**) Postponing, taking little or no food at the beginning (**C**) and Pushing, finishing the food completely in the first 4 h (**D**). All the data were mean ± SD.

An interesting and important fact was achieved in our study that each beagle had different performances for the food taking behaviors. It was observed that four types of food taking patterns, named as “4P Modes”, were classified depending on the accumulated food intake each day of each beagle. Firstly, No. 1, 7 and 9 beagles in group “Persisting” had a continuous slow food intake in 12 h, but not exceeding to 500 g with persistent intake of food (Fig. 1A). Secondly, the beagles of No. 3, 5, 8 and 10 were catalogued in one group as “Pulsing”, which were featured by irregular pulsing food intake. Depending on the amount of food intake at different testing time points, group “Pulsing” tended to eat much food during the first 30 min but less than 200 g, after which the beagles displayed a steady food intake and also consumed no food in particular periods (Fig. 1B). Thirdly, three beagles coded as No. 2, 11 and 12 beagles, were named as “Postponing” type which had very little food (less than 250 g) within 8 h, even taking no food at the beginning after food being provided (Fig. 1C). For the fourth group, there were two beagles (No. 4 and 6) taking food over 250 g in the first 30 min, and finishing completely all the food in the first 4 h, which were assigned into group “Pushing” (Fig. 1D).

**Figure 2:**
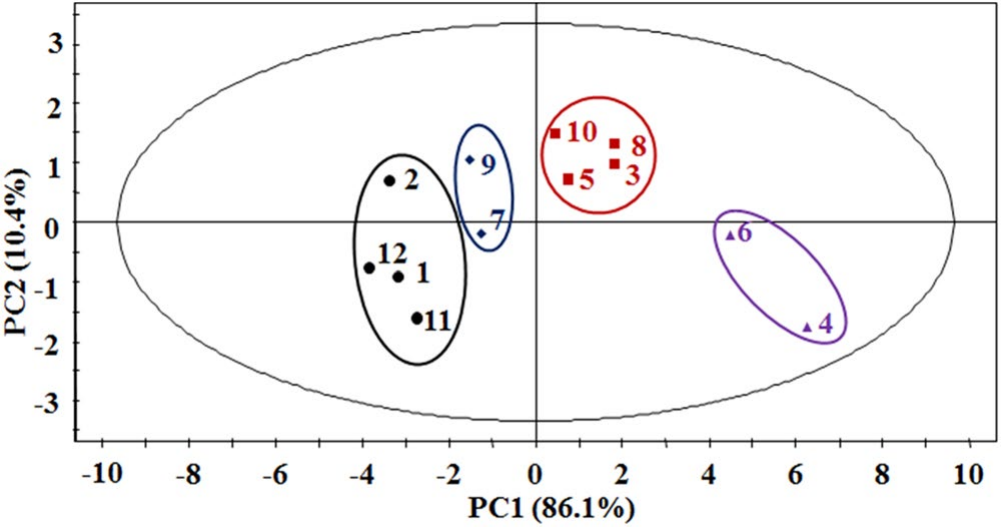
Classifications of food taking patterns for 12 beagles depending on the PCA method. For the score plot, PC1 explained variability X 86.1%, PC2 explained variability X 10.4%.

The average data of food intake in 7 continuous days of beagles were analyzed by PCA, which was to distinguish and separate groups of data effectively (Fig. 2). In PCA analysis, the first principal component (PC1) explained the largest variation, so as the second principal component (PC2) explained the second largest variation. The positive (+) and negative (−) sides of the PC1 and PC2 were separated by the origin. The different samples placed on the same side (positive or negative side) of the PC origin were directly correlated, whereas those located on the different sides of the PC origin were inversely correlated. Furthermore, the samples located near the origin of the PC axes had little or no significant contribution to the optimized model in the selected design space^23^. In this study, the model employed two principal components to explain 96% of the variation in total data matrix. The PC1 had the most significant variability of the data with 86.1%. Meanwhile, this value for PC2 was 10.4%, which contained the limited information for the samples. The PCA score plot showed four distinct groups (No. 1, 2, 11, 12 lower than −2; No. 7, 9 range of −2~0; No. 3, 5, 8, 10 range of 0~2; No. 4, 6 higher than 4) based on the distribution of beagles on the PC1, of which were placed on the same space. Furthermore, the clustering reflected the different food taking patterns of beagle due to the consumption of food as the critical factor in PCA (Fig. 2). Compared with the feeding patterns, the beagles had the same classification, group “Persisting” (No. 7, 9), “Pulsing” (No. 3, 5, 8, 10), “Postponing” (No. 2, 11, 12), “Pushing” (No. 4, 6), except that No.1 beagle was classified as the “Postponing” group depending on the score plot because of its persistent lower food intake.

### Trajectory variation of food intake in inter-day

**Figure 3:**
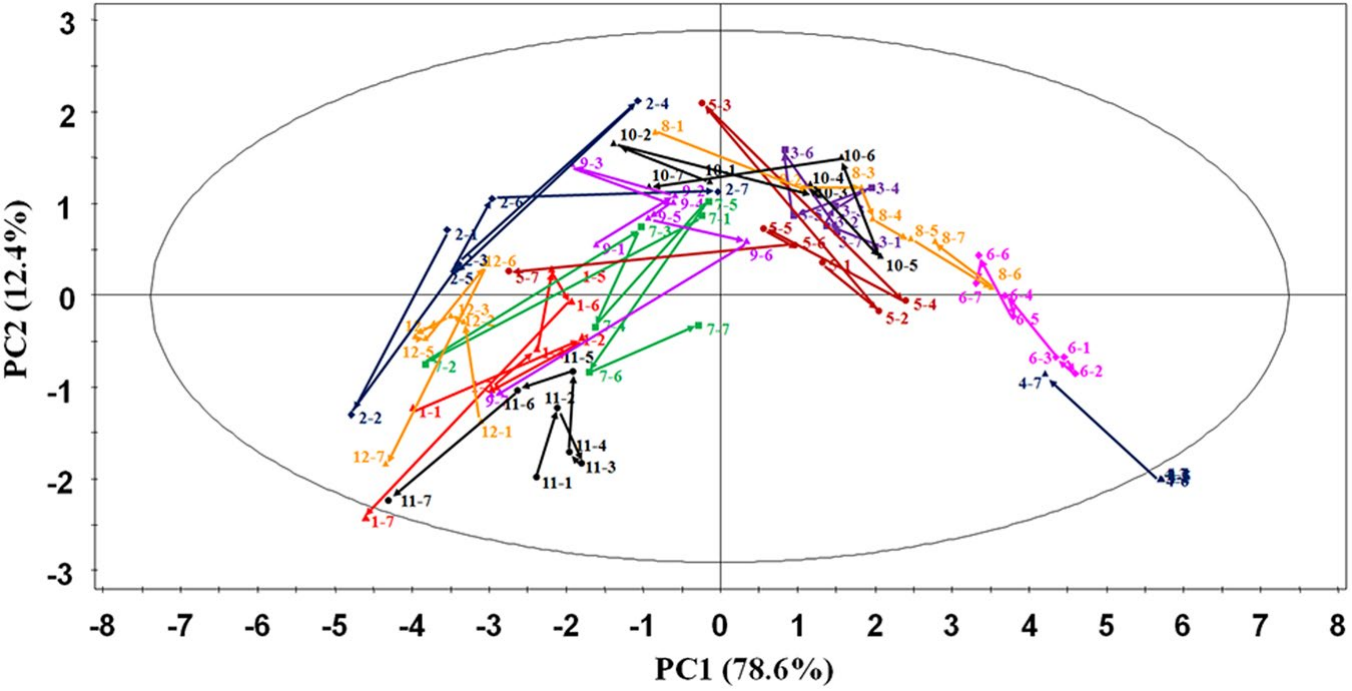
Food taking patterns trajectories of 12 beagles in 7 continuous days uncover food taking patterns variation to each beagle. For the score plot, PC1 explained variability X 78.6%, PC2 explained variability X 12.4%; “i,j” represented the food intake of No. i beagle on the j^th^ day.

The trajectory of food taking patterns in the scores plots could directly demonstrate the inter-day variation of food taking behaviors. The clustering and tendency of the intake food amount in different days represented the degree of stability of food intake in beagles. Herein, all the data of food intake of each beagle in 7 continuous days were analyzed by PCA trajectory (Fig. 3). It was indicated that some beagles took food in a regular way all the 7 days, such as No. 3, 4, 6, 8, 11 and 12, which were more likely to be aggregated when the outliers were ignored, as well as variation of trajectory was in a small scope. On the contrary, some ones irregularly took food during the test, such as No. 1, 2, 5, 7, 9 and 10, which showed some changes in 7 days and did not form obvious clusters. Therefore, the accompanying statistics of the quantities of food intake should be acquired during pharmacokinetic study to exclude the interference of irregularities in the food taking patterns variation.

### Impact of food taking patterns on valsartan bioavailability

**Figure 4:**
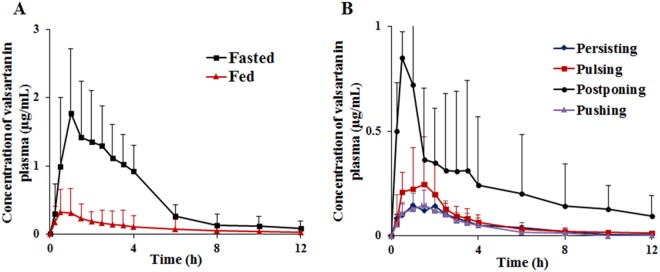
Plasma concentration-time profiles of valsartan capsules varied by food taking in beagles. Fasted and fed states (n = 12). (**A**) “4P Modes” after a single oral dose of 80 mg valsartan per beagle (**B**).

As for the pharmacokinetic study, beagles were orally administered at dose of 80 mg valsartan and the mean plasma concentration-time profiles of valsartan were obtained for the fasted and fed group (Fig. 4A). The corresponding pharmacokinetic parameters were calculated by non-compartment model (Table 1) and it showed that the AUC_0–12h_ of fasted group (6.58 ± 2.45 mg/L · h) was nearly five-fold higher than the fed group (1.22 ± 1.60 mg/L · h). Meanwhile, the fasted group showed a higher C_max_ (2.00 ± 0.77 mg/L) compared to the fed group (0.42 ± 0.37 mg/L). Furthermore, valsartan in fed state resulted in 7.6-fold higher apparent clearance (CL/F), and 1.8 times shorter apparent half-life (t_1/2_) than those in fasted state.

**Table 1:** Mean pharmacokinetic parameters for valsartan capsules after a single oral administration in beagles of 80 mg valsartan per beagle (n = 12).

Parameters	Fasted state	Fed state
AUC_0–12h_ (mg/L · h)	6.58 ± 2.45*	1.22 ± 1.60
T_max_ (h)	1.75 ± 0.99	0.98 ± 0.63
C_max_ (mg/L)	2.00 ± 0.77*	0.42 ± 0.37
t_1/2_ (h)	1.92 ± 0.97	3.48 ± 1.77
CL/F (L/h/kg)	1.10 ± 0.37*	8.34 ± 3.68

Data were reported as mean ± SD. Comparison between groups was done by t-test. *p < 0.05, compared with fed state.

The pharmacokinetic profiles of valsartan plasma concentrations in animals with “4P Modes” were compared (Fig. 4B). It was obvious that the beagles of “Postponing” group was the highest for absorption rate, which reached highest drug plasma level after 0.5 h of administration, followed by a rapid decrease within 2 h. In addition, the AUC_0–12h_ (2.98 ± 2.89 mg/L · h) and C_max_ (0.96 ± 0.32 mg/L) of “Postponing” group was about four-fold higher than that in the other three groups (Table 2), whereas the beagles had slower speed of food taking and less feeding quantity. Furthermore, plasma levels of “Pulsing” group were higher than the “Persisting” group and “Pushing” group, which took 1 h to reach C_max_ level, and then eliminated slowly.

**Table 2:** Pharmacokinetic parameters of valsartan capsules in beagles catalogued into four types in fed state of 80 mg valsartan per beagle.

Parameters	Food taking patterns-“4P Modes”			
	Persisting	Pulsing	Postponing	Pushing
AUC_0–12h_ (mg/L · h)	0.60 ± 0.14	0.81 ± 0.26	2.98 ± 2.89*	0.53 ± 0.04
T_max_ (h)	1.08 ± 0.88	1.13 ± 0.75	0.67 ± 0.29	1.00 ± 0.71
C_max_ (mg/L)	0.19 ± 0.03	0.32 ± 0.18	0.96 ± 0.32*	0.17 ± 0.04

Data were reported as mean ± SD. Comparison between groups was done by t-test. *p < 0.05, “Postponing” group was compared as the reference with other three groups.

### Impact of food taking patterns on valsartan absorption

**Figure 5:**
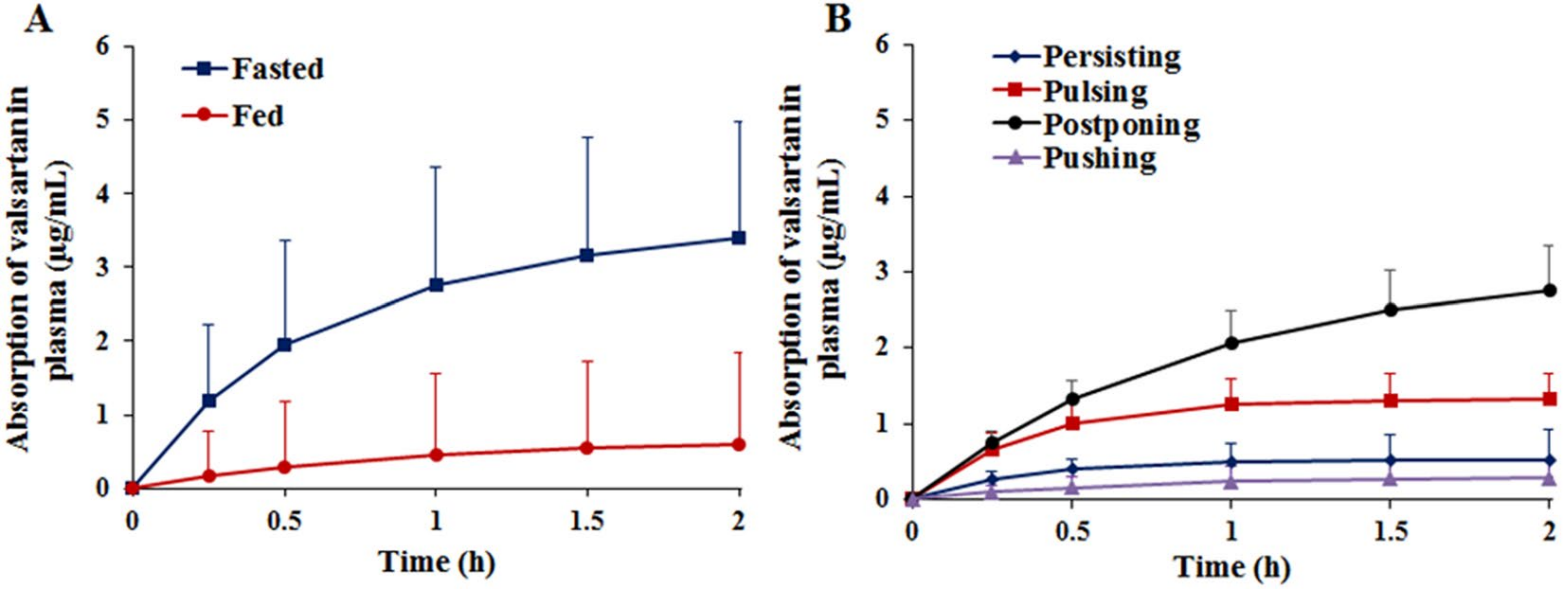
Absorption-time profiles of valsartan capsules varied by food taking in beagles. Fasted and fed state (n = 12). (**A**) “4P Modes” after a single oral dose of 80 mg valsartan per beagle (**B**).

The rate of drug absorption could be affected and altered by fed state. The absorption profiles of the valsartan capsule were obtained by the Wagner-Nelson method^24^ (Fig. 5). The results indicated that the absorption rate in fasted state was obviously higher than that in fed state (Fig. 5A). Furthermore, the absorption profiles of valsartan varied among the “4P Modes”, while the drug absorption in the “Postponing” group was faster than that in the other three groups (Fig. 5B).

### Relationship between food taking patterns and bioavailability

**Figure 6:**
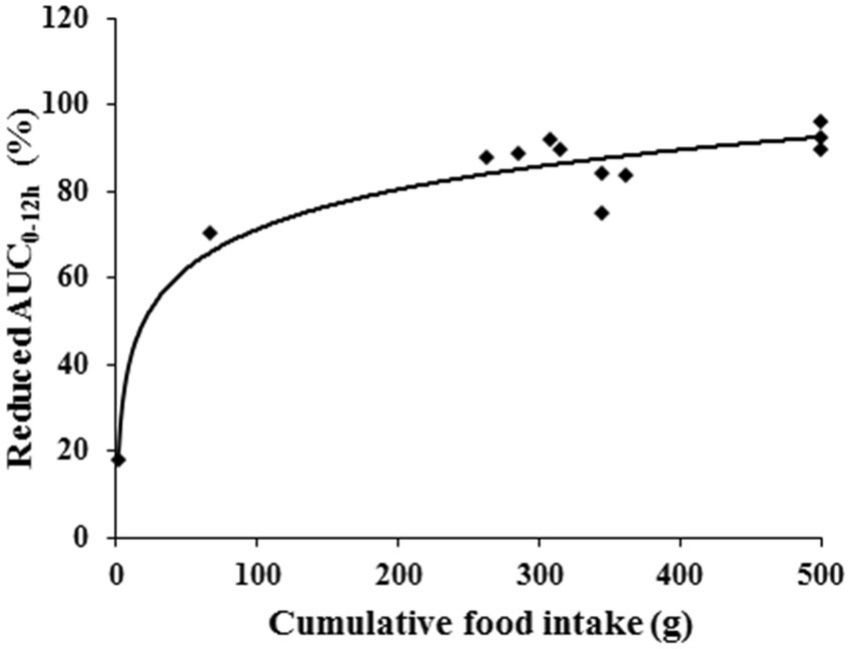
Relationship between cumulative food intake within 8 h with the percentage of AUC_0–12h_ reduction.

The AUC_0–12h_ of valsartan after administration were decreased 86.0% under the fed condition in beagles, while decreased more than 90.3% when the food intake reached 500 g. The percentage of the reduced AUC_0–12h_ was correlated with the logarithm of the food quantity consumed by beagles at the same day with trail of pharmacokinetics (R^2^ = 0.9366) (Fig. 6), indicating that food intake was logarithmically positive correlation with the reduction of bioavailability.

## Discussion

Guidance from the FDA has promoted a serious research process in the food effect on oral bioavailability of drugs under fasted and fed treatment in humans^14^. Food effect on pharmacokinetics is generally conducted through clinical trials directly in the previous reports^25,26^. However, preclinical pharmacokinetic study of drugs in animals is one of the primary parts of drug development processes relevant to accurate evaluation for the dosing, pharmacodynamics and toxicity^27–29^, which can reduce the risk of clinical trials in humans^30,31^. For instance, fenofibrate, nifedipine and sarpogrelate were investigated by food effect in rat, pig and beagle, respectively, which were instead of human^6,17,32^. In this investigation, the beagle as an animal model were employed to evaluate the correlation between the pharmacokinetic parameters (under fasted or fed treatment) and the food taking patterns in detail, which acquired the reliable data for the pharmacokinetics of food effect in preclinical studies, and provided a vital reference for pharmacokinetic study in human.

When using animals to investigate the pharmacokinetic study of food effect, one question to consider is what defines a successful judgement. Animals would not eat accurate quantity of food punctually as required like human, whiles it was usually ignored in the study of food effect^19,33,34^. The beagles might interestingly have different dietary habits which possibly introduced dramatic deviation for the performances of pharmacokinetics experiment designs of food effect. Thus, it was highly significant to understand the food taking patterns of each beagle so as to design rationalized study for the food effects on drug absorption (Fig. 1).

Beagle feeding habits are possibly formed by feeding at the same time point and food quantity every day^35^. Therefore, the food quantity consumed by beagles at different testing time points should be acquired continuously for the verification of food taking patterns. Then the patterns were classified based on the accumulated food intake each day of each beagle (Fig. 1), of which the “4P Modes” was named depending on the different feeding behaviors of the beagles without impact of the weight (Supplemental Table 1). Therefore, the commonly recognition of high body weight meant a high intake of food was excluded. Whereas PCA analysis was also employed to classify the food taking patterns even the amount of food intake was a single factor for the calculation (Fig. 2). Namely, the distribution of beagles on the PCA for the food intake profiles represented the same food taking pattern when they were in the same space. PCA is a widely used statistical technique for unsupervised dimension reduction and visualization of general clustering, such as the field of zoology^36^ and drug researches^37,38^. Above all, it represented valid classification of this study that the similar results of beagle food taking patterns were identified by different methods based upon the feeding behaviors of beagles.

Valsartan is a strongly pH-dependent solubility drug, of which the absorption window is stomach and upper part of small intestine. Thus, absorption rate of valsartan could be decreased when it is taken with food in advance, as well as the exposure of valsartan is reduced^39^. For insights of effects to bioavailability of drugs by food, data sets for oral valsartan administered either fasted or fed were used for pharmacokinetic modeling. A distribution compartment was added when it was necessary to achieve the best fit. One compartment model showed a better fit than a two-compartment model (AIC = −17.6 and −8.1, respectively) for valsartan in fasted beagles. However, a two-compartment model provided a better fit to the data than a one-compartment model (AIC = −69.5 versus −18.8) for valsartan in fed beagles. The compartment models were revealed due to the difference of pharmacokinetics parameters such as apparent clearance (CL/F) and apparent volume of distribution (V/F) between fed and fasted state, which could lead to altering the drug exposure *in vivo*.

As expected, the food taking patterns could significantly affect the pharmacokinetics parameters of valsartan in beagles. “Postponing” type, which had very little or no food within 8 h after food being provided (Fig. 1C), was significantly higher than the other three groups of the plasma levels of valsartan (Fig. 4B), but was close to the fasted group. This was mainly affected by the absorption rate (Fig. 5B), and confirmed by the Wagner-Nelson method, whereas the past reports expressed AUC instead of the absorption^40^. Therefore, food taking patterns (“4P Modes”) caused volumes of food varied in the stomach and the intestine, resulting in the bioavailability of valsartan varied in beagles. Generally, the potential effects included delaying gastric emptying, stimulating bile flow, changing the pH of GI, increasing splanchnic blood flow and changing luminal metabolism of a drug substance and physically or chemically interact with nutrients^41^. In order to investigate the effect of pH in GI, the new formulation was prepared with the solution enhanced about two-fold in the simulated gastric juice (pH = 1.2) by supramolecular technique, which had bioavailability also affected by food at same degree with Diovan®, indicating that the pH of GI was not the single influence factor. The physical or chemical interactions between nutrients and valsartan were examined by the co-incubation tests. It was found that there was no physical adsorption between food and valsartan when the nutrients and valsartan were incubated. Furthermore, valsartan was excreted in the original form, which could exclude the factor of change luminal metabolism. However, most of the orally administered dose of Angiotensin Receptor Blockers (ARBs) were excreted via bile into the faeces, stimulated bile flow was a possible factor for the AUC_0–12h_ reduced by food effect^21,42^. An interesting fact was that percentage of AUC_0–12h_ reduced was correlated with the positive of the food quantity consumed by beagles (Fig. 6) and also proved by PLSDA (Supplemental Fig. 1), which proved that the amount of food intake resulted in the decrease of bioavailability of valsartan. Herein, the right animals with proper food taking patterns should be assessed and selected for food effects evaluation on bioavailability. On the contrary, the results of pharmacokinetics could be discrepancy without screening of beagles. Therefore, it should become a standard practice of preclinical pharmacokinetic study, although many other possible factors should be considered for further research.

The bioavailability study of valsartan showed that “4P Modes” had different degrees of influence on the bioavailability of drugs (Figs 4B and 5B). Individual differences could be obvious in the pharmacokinetics of food effect due to the “4P Modes”. Affectd by “4P Modes” of food taking, nonequivalent results could be obtained for two formulations in bioequivalence study under fed condition^43^. Therefore, the identification of the “4P Modes” was extremely important on the bioavailability study. Even there was a doubt that the effects of food taking pattern could be offset by the self-controlled principle concerning the relative bioavailability, it had been validated that the variation of food intake of beagles in different days (Fig. 3) affected the relative bioavailability involving two-cycle study. Though the “4P Modes” presented in this study of beagles might not be applicable to all kinds of animals or human being, food taking patterns should be identified before the *in vivo* pharmacokinetics evaluation.

In this research, general considerations in the design of experiment for food effect to the bioavailability in beagles were established based on the results. First of all, it was obvious that the food taking patterns of beagle should be evaluated as the initial part of the pharmacokinetics because the bioavailability and absorption rate of valsartan was affected by the “4P Modes” (Figs 4B and 5B). Secondly, it was necessary to confirm the intra-day stability of the food taking patterns of each beagle through a period of time, e.g., one week (Fig. 3). If it was evaluated in a few days, the food taking patterns of beagles were illegible. Beagles were fixed ordinary amount food depending on the growth periods of them, so food was given 500 g/day to beagles in this study. Thirdly, the feeding time should be controlled at the same time with pharmacokinetic sampling time to re-confirm the food intake patterns during plasma sampling (Figs 1 and 4). Finally, the right animal with proper food taking patterns should be chosen after food taking behavior classification.

In summary, the bioavailability of drugs in animals with fasted and fed conditions should be determined to assess the effect of food on pharmacokinetics for the orally administered drugs. It is assessed by this research that each animal has different food taking behaviors and the food taking patterns may vary in different days. This research helps to clarify the key issues in the food effect of preclinical pharmacokinetic study. The beagles show “4P Modes” of food taking patterns, namely, Persisting, Pulsing, Postponing, Pushing styles, which change or shift more or less with inter-day variations. As demonstrated in the study of valsartan, parallel tracing of the food taking patterns along with plasma level determination has successfully assessed the significance for the identification of beagle food taking patterns to the establishment of a protocol for the evaluation of food impacts on bioavailability.

## Materials and Methods

### Materials

Valsartan capsules (Diovan®, batch number: X1631, specifications: 80 mg) were purchased from Beijing Novartis Pharmaceutical Co., Ltd. Valsartan reference material (Purity ≥98.5%, National Institutes for Food and Drug Control, China) and internal standards (IS) of hydrochlorothiazide (Purity ≥99.0%, Liaoning Province Institute of Food and Drug Control, China) were used for preparation of standard stock solutions. Formic acid (LC-MS grade) was purchased from Sigma-Aldrich (Shanghai, China). Methanol and acetonitrile (LC-MS grade) were obtained from Merck (Shanghai, China). Ultrapure water (18.2 MΩ) was prepared with a Milli-Q water purification system (Millipore, Germany).

### HPLC-MS/MS conditions

Chromatographic analysis was performed on an HPLC system (Agilent, 1260, USA), including a binary pump solvent manager (G1312C), a column oven (G1316A) and an autosampler (G1367E). Valsartan and IS were separated on an core-shell XB-C18 column (150 mm × 4.6 mm, 5 μm, Phenomenex, USA) at 35 °C. The mobile phase consisted of 0.1% formic acid aqueous and acetonitrile (40:60, v/v), which was performed at the flow rate of 0.6 mL/min. An injection volume of 5 µL was applied for analysis.

The triple-quadrupole tandem mass spectrometric detection (Agilent, G6460A, USA) operation parameters were obtained and optimized under negative-ion mode (Electrospray ionization, ESI^-^, G1958-65138). The optimal MS parameters were as follows: capillary voltage, 3.5 kV; nebulizer, 35 psi; sheath gas temperature, 350 °C; drying gas temperature, 300 °C; both of sheath and drying gas flow were 10 L/min. The fragmentation transitions for the multiple reaction monitoring (MRM) were m/z 434.1 → 179.1 for valsartan and m/z 295.9 → 268.8 for the hydrochlorothiazide, respectively. All data were acquired by MassHunter V4.1 software (Agilent, USA).

### Sample preparation

Plasma samples were removed from −80 °C storage and placed under ambient conditions. Aliquots of 100 µL for the plasma samples were spiked with 25 µL of hydrochlorothiazide (100 ng/mL) and 25 µL of solution (acetonitrile:water, 60:40, v/v), and then vortexed for 30 s. Then, aliquots of 350 µL acetonitrile were added and vortexed for 30 s. After mixing, the samples were centrifuged at 12,000 rpm for 5 min. The supernatant of 5 µL were injected into HPLC-MS/MS for analysis.

### Animals

Twelve adult beagles (weighing 11~18 kg, 6 males and 6 females) were obtained from the Agricultural College, Shanghai Jiao Tong University. The animals were housed under the condition of sustained temperature (25 ± 1 °C), humidity (~60%) and a 12 h light/dark cycle. All procedures involving animals were performed in accordance with the Code of Ethics of the World Medical Association and approved by the Institutional Animal Care and Use Committee of Shanghai Institute of Materia Medica, Chinese Academy of Sciences (IACUC application №: 2017-05-ZJW-12).

### Experiment design

This study was a two-treatment, randomized crossover study conducted in healthy beagles with a two-week washout period between treatments. Twelve beagles were randomly divided into the fasted group and the fed group. Both of the two groups were orally administrated with valsartan capsules at the dose of 80 mg. For the fasted group, the beagles were fasted for 12 h with free access to water prior to the experiments, and then provided a unified food (500 g dog food) after 4 h of the drug administration. For the fed group, dog food of 500 g was provided to each beagle before 30 min of the drug administration. Blood samples (about 1.5 mL) were collected into heparinized centrifuge tubes via the forelimb vein at 0, 0.25, 0.5, 1, 1.5, 2, 2.5, 3, 3.5, 4, 6, 8, 10, 12 h after administration. During blood collecting, the remaining dog food for each beagle was also weighed at the same time. Plasma samples were centrifuged for 10 min at 4,000 rpm. After centrifugation the plasma was separated and then stored at −80 °C until analysis.

### Study of food taking patterns

For the comprehensive and detailed study of food taking patterns in beagles, the food taking was carefully measured in the 7 continuous days by weighing the remaining food, so as to calculate the quantity of consumed food. The time point was the same as the pharmacokinetic study.

### Data analysis

The obtained data were analyzed using PCA and PLSDA (SIMCA-P+ 11.0 software, Umetrics, Sweden). The pharmacokinetic parameters of analytes were calculated using the DAS 2.0 software as mean ± standard deviation (SD) and examined by t-test. All statistical analyses were performed with SPSS 17.0 (SPSS Inc., Chicago, IL, USA). P-values of *p* < 0.05 was considered to be statistically significance.

## Electronic supplementary material

Supplementary results
