# Supplementary material for: Identification of beagle food taking patterns and protocol for food effects evaluation on bioavailability
Source: Sci Rep. 2018 Aug 24;8:12765. doi: 10.1038/s41598-018-30937-1 (PMC6109188; doi:10.1038/s41598-018-30937-1)
Supplement: Supplementary file 1 — Supplementary results [file 41598_2018_30937_MOESM1_ESM.doc]

**Supplemental material**

**Identification of beagle food taking patterns and protocol for food effects evaluation on bioavailability**

Authors:

Guoqing Zhang^1,2,*^, Caifen Wang^2,*^, Li Wu^2,3^, Jian Xu^2,4^, Xiaoxiao Hu^2^, Shailendra Shakya^2,4^, Yuanzhi He^2^, Xiaohong Ren^2^, Weidong Chen^1^ & Jiwen Zhang^1,2,4^

Affiliations:

^1^Institute of Drug Metabolism, School of Pharmaceutical Sciences, Anhui University of Chinese Medicine, Hefei, China; ^2^Center for Drug Delivery Systems, Shanghai Institute of Materia Medica, Chinese Academy of Sciences, Shanghai, China; ^3^School of Pharmacy, Key Laboratory of Molecular Pharmacology and Drug Evaluation, Ministry of Education, Yantai, China; ^4^University of Chinese Academy of Sciences, Beijing, China. ^*^These authors contributed equally to this work. Correspondence and requests for materials should be addressed to J.Z. (jwzhang@simm.ac.cn) and W.C. (anzhongdong@126.com).

**Relationship between food taking patterns and bioavailability.** In order to establish the relationship between the food intake and reduced AUC, all the data of food intake including 7 days and all the time point of plasma sampling between fed and fasted state were analyzed by PCA. Furthermore, partial least square discriminant analysis (PLSDA) was applied to examine the same data. The PCA analysis (Figs. S1A, S1B) illustrated that both of food intake and concentration of valsartan had the high influence on PC1 and PC2 which show variability of 86.1% and 10.4% depending on the score plot, respectively, and showed positive correlation with PC1 and PC2. For the PLSDA analysis (Figs. S1C, S1D), the score plot was different with PCA. However, the two factors had the highest influence on PC1 (59.2%) and PC2 (18.7%), and also showed positive correlations.

**
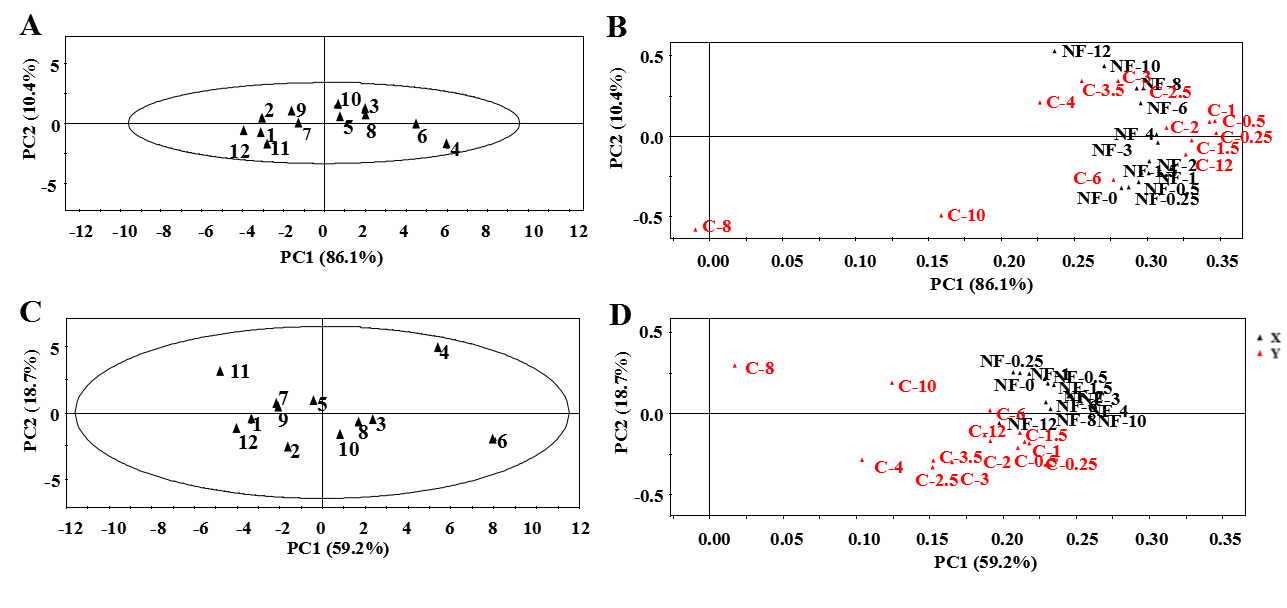
**

**Figure S1. PCA and PLSDA score and loading plot for all food intake (“NF-i”) and concentration of valsartan (“C-i”)**. “i” represented the different time point. Score plot and loading plot of PC1 and PC2 of PCA, PC1 explained variability X 86.1%, PC2 explained variability X 10.4% (A-B); Score plot and loading plot of PC1 and PC2 of PLSDA; PC1 explained variability X 59.2%, PC2 explained variability X 18.7%, respectively (C-D). 1 to 12 represent No.1 to No.12 beagles.

**The weight of each beagle.**

**Table S1.** **The weight of each beagle of different types.**

| Types | Number | Weight (kg) |
| --- | --- | --- |
| Persisting | 1 | 15.42 |
|  | 7 | 14.89 |
|  | 9 | 13.26 |
| Pulsing | 3 | 17.59 |
|  | 5 | 13.28 |
|  | 8 | 14.66 |
|  | 10 | 13.53 |
| Postponing | 2 | 15.78 |
|  | 11 | 11.28 |
|  | 12 | 11.35 |
| Pushing | 4 | 15.28 |
|  | 6 | 13.28 |
